# No evidence for assortative mating within a willow warbler migratory divide

**DOI:** 10.1186/s12983-014-0052-2

**Published:** 2014-07-12

**Authors:** Miriam Liedvogel, Keith W Larson, Max Lundberg, Arzu Gursoy, Leonard I Wassenaar, Keith A Hobson, Staffan Bensch, Susanne Åkesson

**Affiliations:** 1 Center for Animal Movement Research (CAnMove), Department of Biology, Lund University, Lund, 22362, Sweden; 2 Wildlife Ecology and Management, Freiburg University, Tennenbacher Str. 4, Freiburg, 79106, Germany; 3 Department of Biology, Faculty of Arts and Sciences, University of Ondokuz Mayis, Samsun, Turkey; 4 Environment Canada, 11 Innovation Blvd., Saskatoon S7N 3H5, SK, Canada

**Keywords:** Hybrid zone, Prezygotic selection, Postzygotic selection, Reproductive isolation, Willow warbler, Phylloscopus trochilus, Nitrogen-15

## Abstract

**Introduction:**

In contact zones, genetic mixing of two taxa can be restricted by prezygotic (e.g. assortative mating) or postzygotic (lower fitness of hybrid offspring) barriers, or a combination of the two. A hybrid zone between two willow warbler subspecies (*Phylloscopus trochilus trochilus, P. t. acredula*) with distinctive migratory strategies occurs in central Sweden. These subspecies exhibit differences in migratory direction and distance, resulting in geographically distinct wintering areas in Africa. The subspecies may have diverged from a common refuge after the last ice age, and neutral genetic markers are homogeneous across their range. By contrast, several phenotypic traits and genetic markers of two chromosomal regions previously identified show steep clines across the divide. The evolutionary forces that maintain this migratory divide remain unknown. Here we use plumage colour, morphology, genetic markers and feather stable nitrogen-isotopes (*δ*^15^N) to assess if assortative mating between migratory phenotypes could be acting as a possible mechanism for keeping the two forms genetically separate and maintaining the migratory divide. We colour-ringed a willow warbler breeding population in the central part of the hybrid zone and observed the breeding population to assess phenotypic and genotypic traits of social pairs.

**Results:**

Our data suggest that wintering area and genetic ancestry had an effect on male arrival time to the breeding grounds which could contribute to assortment. However, evidence for assortative mating could not be detected based on a comparison of plumage colour, morphology and *δ*^15^N between social mates.

**Conclusion:**

This finding was strengthened by analyses of subspecies-specific genetic markers, which allowed us to identify the presence of a large proportion of potential hybrids and backcrosses at the study site. Our results supported the hypothesis that pre-mating isolation in willow warblers is weak, resulting in extensive hybridisation across the migratory divide.

## Introduction

Migratory divides are narrow regions of parapatry where neighbouring populations of closely related taxa, but with distinctly different migratory behaviour, meet and potentially hybridise [[1]-[3]]. Divides may result from dispersal and range expansion of two former allopatric populations that over time meet in secondary contact [[4]]. Examples of migratory divides based on secondary contact include the European blackcap (*Sylvia atricapilla*) [[5]] and Swainson’s thrush (*Catharus ustulatus*) [[6]]. In addition, migratory divides could also be established if an alternative migratory phenotype, which may differ in migratory direction, distance to wintering grounds or the propensity to migrate, becomes selectively beneficial and therefore increases in frequency. Given strong selection pressures, the frequency of the alternative migratory phenotype could increase within a small number of generations. This has been seen in European blackcaps, for which a new major migratory direction to wintering areas in Britain and Ireland evolved over the past fifty years [[7],[8]]. From quantitative genetics analyses we know that a significant proportion of migratory trait variation is genetically controlled (reviewed in [[9]]), e.g. encoding migratory direction, as well as an inherited time schedule that determines departure and arrival time (i.e. defining a migratory distance). This inherited time schedule further coordinates the temporal expression of physiological adaptations necessary for a successful migratory journey, such as post-breeding moult and pre-migration fattening [[10],[11]].

Assortative mating provides an important mechanism for reproductive isolation between parapatric populations [[12],[13]]. Differences in habitat choice, diet, breeding phenology and song and/or mating behaviour may all contribute to the establishment of prezygotic reproductive barriers and specifically assortative mating [[12],[14],[15]]. For example, in the European blackcap, the timing of arrival to the breeding grounds promotes assortative mating between two co-occurring migratory phenotypes with separate wintering grounds in the UK and the Iberian Peninsula to NW Africa [[14],[16]]. Similarly, postzygotic selection may result from maladaptive combinations of intermediately inherited migratory traits in hybrids. In cross-breeding experiments with European blackcaps with parents from either side of a migratory divide, F_1_ hybrid offspring demonstrated an intermediate migratory direction compared to the parental phenotypes [[17],[18]]. In the wild, this intermediate route may be selected against, because it necessitates crossing additional significant ecological barriers lacking refuelling and stop-over habitat (e.g. the Alps as well as crossing both the Mediterranean Sea and Sahara desert at their widest stretches) without the necessary physiological adaptations for long-distance flights [[17],[19]].

In central Scandinavia two subspecies of the willow warbler (*Phylloscopus trochilus trochilus* in the south, and *P. t. acredula* in the north) with distinctive migratory behaviour meet and form a migratory divide. Each migratory phenotype likely possesses distinct endogenous migratory traits, including timing, migratory direction and distance, but presumably also different physiological adaptations such as fuelling decisions [[20]]. In southern Sweden *P. t. trochilus* migrates SW in the autumn to winter in West Africa, whereas *P. t. acredula* migrates SSE to winter in East to South Africa. Migratory direction and wintering grounds for the two subspecies are inferred from ringing recoveries [[1],[21],[22]] and stable nitrogen-isotope analyses (*δ*^15^N) of winter-grown feathers [[1],[23]].

Molecular analyses using neutral markers (microsatellites, mtDNA) reveal no intraspecific population structure (F_ST_ ≈ 0) [[19],[22]]. This may be explained by a very recent divergence between the subspecies [[19],[22]]. In contrast to the high level of genetic similarity, phenotypic traits including plumage colouration, morphology, and *δ*^15^N (proxy for migratory direction and wintering grounds) and a genetic marker associated with migratory direction (identified by AFLP scan), present steep clines across the migratory divide (<300 km) [[1],[19]].

Previous work demonstrated that the central Scandinavian migratory divide is maintained by selection, as geographic clines for both phenotypic and genotypic traits are narrower than expected from a neutral diffusion model assuming random mating between the subspecies since the time of secondary contact [[19],[22]]. Random mating could be one important contributor to this; the more mating is assortative, all other factors being equal, the wider the cline will be. However, geographic clines of mean trait values across different localities do not explicitly provide information on the degree of hybridization that is associated with intermediate trait values and allele frequencies across the migratory divide. The proportion and types of hybrids is determined by post-zygotic selection (reduced fitness in hybrids) and degree of assortative mating between the parental forms: the more post-zygotic and less pre-zygotic selection present, the narrower the hybrid zone.

We quantify the proportion and different classes of hybrid genotypes in the migratory divide. We also assess to what extent assortative mating is occurring and how it is related to the observed degree of hybridization.

We addressed this question by studying a breeding site in the centre of the hybrid zone where we expected both subspecies to occur. We assessed the degree of assortative mating based on a comparison of the characteristic phenotypic traits for pairs captured on their breeding territories. In order to estimate the genetic ancestry of individuals, we made use of subspecies-specific genetic variation segregating at two newly identified loci that had been detected through analyses of transcriptome sequencing data [[24]]. These co-dominant markers enabled us to estimate the genetic ancestry in the willow warbler at a much higher accuracy than with the AFLP markers. This data improved our quantification of potential hybridisation and allowed for detection of more complex hybrids and backcrosses.

The key objectives of our study were to identify evolutionary processes and selective forces within our focal breeding population by (i) characterising the distribution of phenotypic and genotypic traits in a breeding population of willow warblers in the hybrid zone, in order to subsequently (ii) assess the role of prezygotic selection in maintaining the migratory divide.

## Results

### Sampling

We captured individuals of 25 breeding pairs in 2011, and 15 breeding pairs in 2012. In addition, we captured 13 additional males holding territories in 2011, and 22 in 2012. In 2012, late winter weather delayed spring arrival and breeding, which accounted for the lower number of breeding pairs captured.

### Genetic analyses

**Figure 1:**
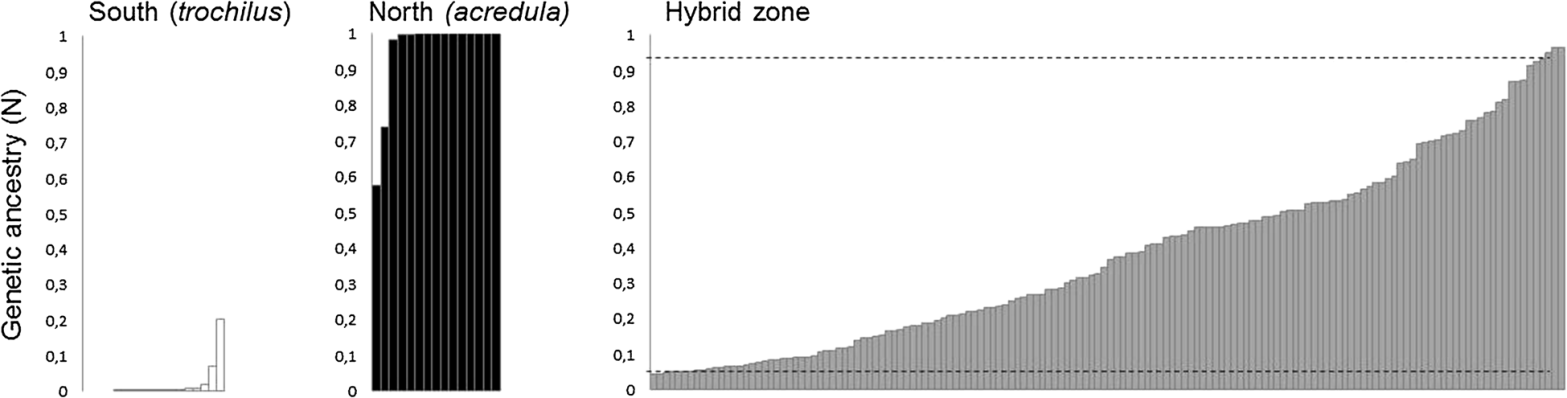
**Genetic ancestry based on two subspecies-specific markers.** Bayesian assignment probability (genetic ancestry) to a northern (*acredula*) subspecies population cluster for all individuals computed in STRUCTURE (n = 181; details listed in Additional file 1: Table S2). Reference samples for the southern (*P. t. trochilus*, n = 18, open bars) and northern (*P .t. acredula*, n = 15, black bars) subspecies were plotted separately from the samples collected in the hybrid zone (n = 148, grey bars). Each bar represented one individual; the y-axis represented the probability of genetic ancestry to the northern subspecies cluster. The distribution within the study population in the hybrid zone was skewed towards southern genotypes. Dashed lines in the hybrid zone population indicated mean ancestry estimates for individuals from the northern and southern reference sets, respectively.

With few exceptions, individuals in both subspecies-specific reference sets were assigned to each of the two population clusters with high probabilities using the newly identified subspecies-specific genetic markers (Figure 1, Additional file 1: Table S2). This suggested that both identified population clusters corresponded to each of the subspecies. The few individuals in each of the reference sets that obtained a lower assignment probability to the expected subspecies cluster possibly indicated a hybrid origin. Individuals at the study site showed much higher variability in genetic ancestry compared to sampling areas at either side of the divide outside the hybrid zone (Figure 1, Additional file 1: Table S2). In general, ancestry estimates were skewed towards the southern subspecies, with a declining proportion of individuals with increased ancestry to the northern subspecies (Figure 1, Additional file 1: Table S2). The number of individuals from the study site that were likely to be pure individuals of the northern subspecies was very small.

### Arrival time

**Figure 2:**
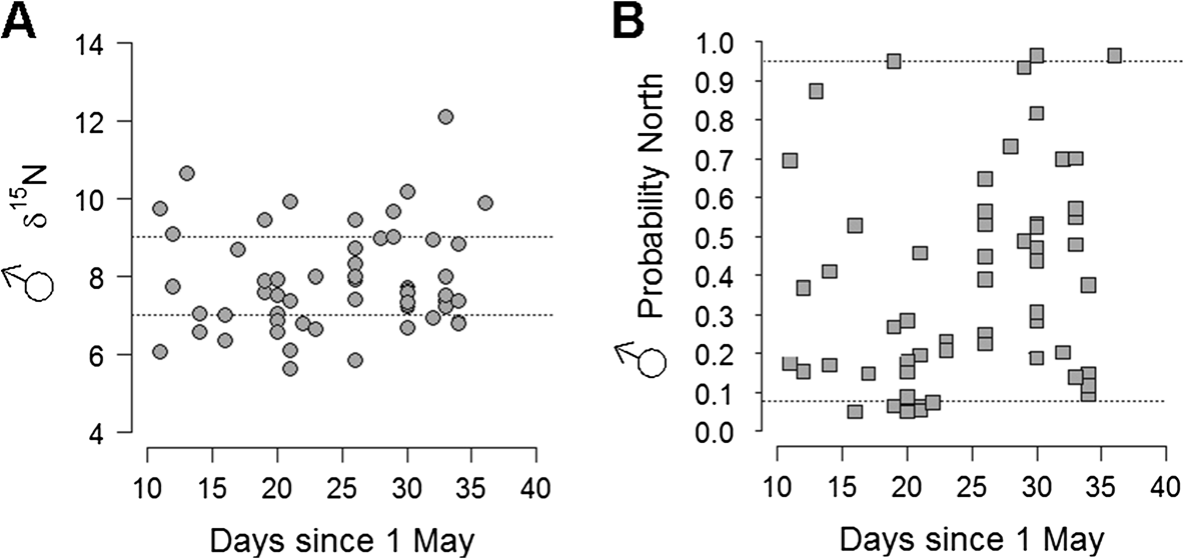
**Male phenotype (A) and genotype (B) in relation to capture date (for males captured between 10 May and 10 June) (n = 56).A***δ*^15^N values (dashed lines at 7‰ and 9‰ represented cut-offs for individuals likely wintering in West Africa and East to South Africa, respectively), **B** Bayesian assignment probabilities (genetic ancestry) to the northern subspecies cluster (dashed lines represent mean assignment probabilities in the southern and northern reference sets, respectively). Time for capture dates was plotted as the number of days after 1 May.

During the period of intense field work we were confident that most of the encountered unringed birds were newly arrived to our study area. We thus expected capture date and arrival date to be correlated for this interval. If timing contributes to assortative mating in the hybrid zone, we also expect birds from different wintering grounds (indicated by *δ*^15^N feather values) to arrive at differing schedules. We found no significant correlation of male *δ*^15^N feather values with capture date in males (*r* = 0.15, *n* = 54 *p* = 0.14) (Figure 2A). However, there was a positive correlation between capture date of males and genetic ancestry, measured as probability of the individuals belonging to the northern subspecies cluster (*r* = 0.24, *n* = 54 *p* = 0.04) (Figure 2B).

### Assortative mating

**Figure 3:**
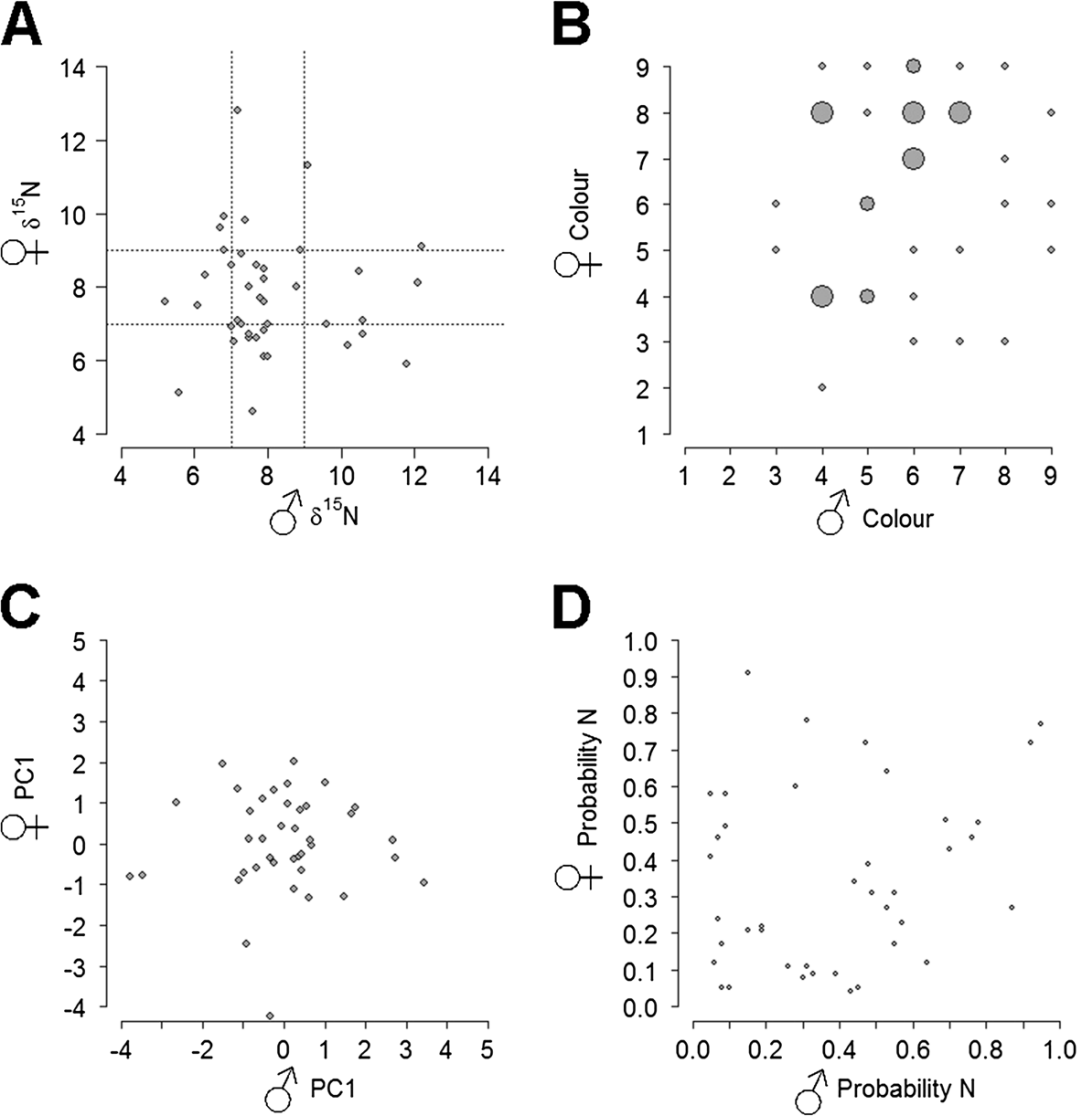
**Male–female trait comparison for all pairs (n = 40).A***δ*^15^N values; dashed lines at 7‰ and 9‰ represented cut-offs for birds likely wintering in West Africa and East to South Africa, respectively; **B** plumage colour scores; **C** PC1 for body size; and **D** genetic ancestry to the northern subspecies. Pair counts for colour and genetic ancestry (values rounded to 2 significant digits) were represented by the size of the circle.

We tested for assortative mating for 40 confirmed pairings based on comparisons of phenotypic values using Pearson’s correlations (see Additional file 2: Table S4 for all pair trait data). Overall, within-pair comparisons of the three phenotypic traits revealed no evidence for assortative mating. First, for *δ*^15^N values (indicator of wintering grounds) there was no relationship between the values of the social mates (*r* = −0.03, *n = 38*, *p* = 0.58) (Figure 3A). Likewise plumage colour did not show a clear correlation between social mates (*r* = 0.23, *n = 38*, *p* = 0.12) (Figure 3C). For body size as summarised by PC1 (*r* = 0.02, *n = 37*, *p* = 0.46) (Figure 3D) and PC2 (*r* = −0.14, *p* = 0.80) there was no relation between the values of the social mates. Body size PCA results and trait values for all social pairs are summarised in the Additional file 1: Tables S1 & S2. No significant correlation (*r* = 0.22, *n = 38*, *p* = 0.08) could be detected between the genetic ancestry of social mates.

## Discussion

Genetic ancestry for all males was significantly correlated with arrival date. Although we did not have data on female arrival, it is known from other studies that male willow warblers arrive on the breeding ground one to two weeks ahead of females (e.g. [[25]]). The large overlap in arrival time of the males of mixed genetic ancestry combined with females presumably arriving substantially later than the males, suggested that differential arrival time played a limited role in assortative mating in willow warblers. This is in contrast to the pattern found in populations of European blackcaps, where two populations with distinctly different migratory strategies breed in sympatry, but mate assortatively based on timing of arrival to the breeding grounds [[14]] as has also been observed in, Swainson’s thrushes [[26]] across their migratory divide. In both cases differences in arrival time at the breeding grounds is a major force for assortative mating. In willow warblers birds of the northern subspecies arrive later. However, our data did not suggest assortative mating based on the difference in arrival time of both subspecies. Research in blackcaps suggests that hybrid phenotypes have an intermediate endogenous migratory programme compared to the parental phenotypes. It is possible that an intermediate strategy might result in reduced hybrid survival [[17]]. Another, and not mutually exclusive, possibility is that a reduction in hybrid fitness is mediated by genetic incompatibilities [[27]]. As the willow warbler subspecies are genetically similar (as is also true for blackcaps), it seemed unlikely that genetic incompatibilities act as selective forces in keeping the hybrid zone narrow.

We further observed no evidence for assortative mating in the hybrid zone based on a comparison of several characteristic phenotypic and genotypic traits. Careful within-pair comparison of morphological traits (plumage colour, body size), genotype, and migratory direction, which all show steep clines across the migratory divide, indicated no pattern of assortative mating between the pairs. A similar pattern has been found in the hybrid zone of yellow-rumped warblers, where extensive hybridisation in the absence of assortative mating occurs [[28]]. Our results suggest that assortative mating in our study system is unlikely to be important in constraining the hybridization across the migratory divide.

Other ecological (e.g. habitat) or sexual (song) traits can be excluded as likely factors in stabilising the willow warbler hybrid zone. This is unlike, e.g. Swainson’s thrushes, where both co-occurring subspecies occupy distinct ecological niches [[26]], or blackcaps, where a recent study provides support for differences in habitat choice but not arrival time being associated with alternative migratory strategies in the populations studied [[29]]. Both willow warbler subspecies breed in similar terrain with trees and shrubs across their range, thus habitat is unlikely to act as barrier in promoting assortative mating in our system. Similarly, song was an unlikely mechanism for assortative mating in willow warblers, as the subspecies’ songs are indistinguishable (unpublished data), and territorial behaviour (i.e. antagonistic reaction to playback of conspecific song) of males across Scandinavia is triggered by the same play-back recording of a conspecific song [[19],[22]], unpublished results]. Also, even if slight song differences do exist but escaped our detection, it would not be possible to evaluate how these differences would result in assortative mating as we have no assay to score female preferences for song in the field. European blackcaps show a similar pattern of symmetrical responses to song playbacks [[29]], but Swainson’s thrushes show an asymmetrical response pattern where one subspecies responds more aggressively to homotypic song whereas the other subspecies’ response pattern to homotypic and heterotypic songs is indistinguishable [[26]].

Based on the SNP data in the two sequenced genes we were able to assign probabilities for each individual to the two population clusters (i.e. northern and southern subspecies) identified by STRUCTURE [[30]]. As expected, individuals in the subspecies reference sets generally showed high assignment probabilities to each of the two clusters, i.e. migratory phenotypes (Figure 1). However, a few individuals in both sets showed a lower assignment probability to their expected population cluster. One possible explanation may be that a small fraction of pure individuals of each subspecies could possess alleles that are more common in the other subspecies at any of the two loci we genotyped. Alternatively, long-distance dispersal may be occurring from the hybrid zone into the parental populations.

The use of only two diagnostic markers was insufficient to detect more complex hybrids, such as advanced backcrosses, which would result in an underestimation of hybridisation. The low number of markers also prohibited reliable classification of individuals into discrete hybrid classes, such as backcrosses, F1 and F2 hybrids [[31]]. Even though our genetic ancestry estimates had some uncertainty and may underestimate the degree of hybridisation, the distribution of ancestries at the study site was markedly different from that seen in the subspecies reference sets (Figure 1). The large proportion of intermediate genetic ancestry estimates at the study site suggested that hybridisation is extensive within the hybrid zone. The skewed distribution of individuals with southern ancestries and the gradual decrease of increasingly northern individuals were expected with extensive hybridisation in the southern part of the hybrid zone. This distribution also suggested that it is unlikely that pure southern and pure northern birds co-occur at high frequencies anywhere in the hybrid zone.

Our sample of 40 pairs of willow warblers were collected over two years from one breeding population in the centre of the migratory divide. The site was carefully chosen because of its location based on previously described trait clines [[19]]. However, our newly established gene ancestry analyses presented here allowed us to identify an excess of birds with *P. t. trochilus* genotypes at the study plot, suggesting that our focal breeding population was situated slightly south of the centre of the migratory divide, if we assumed a smooth cline in genetic ancestry across the divide. The possibility that our study site may indeed be situated south of the divide may have limited the statistical power to detect the possible occurrence of assortative mating. However, none of the characteristic traits investigated showed any correlation between pairs implying that lack of assortative mating was the most parsimonious conclusion for our data.

The lack of assortative mating in willow warblers combined with our previous work further supported the suggestion that postzygotic selection against hybrids rather than prezygotic selection (mate choice) is the major force maintaining this willow warbler migratory divide. The degree of intrinsic reproductive isolation generally accumulates with the genetic distance between the hybridizing taxa [[32]]. In birds, a complete loss of viability and fertility in hybrids is typically seen over millions of years [[33]]. Given the very short time since the divergence between the willow warbler subspecies - estimated to be around 10.000 years [[19]] - severe genetic incompatibilities were unlikely to have evolved. This was also suggested by the high, and yet probably underestimated, amount of potential backcrosses and hybrids at the study site. However, some genetic incompatibilities may only manifest themselves in certain genotypes found in later generation hybrids and backcrosses [[27],[34]]. Our present data set only allowed us to speculate, but not reliably detect such an effect.

## Conclusions and outlook

Our data suggest that without a clear separation in arrival time, habitat choice, song or some other phenotypic traits that would result in assortative mating, hybridisation will remain common throughout the willow warbler migratory divide. Given that mating in willow warblers appears to be random, future research will focus on understanding the mechanisms governing postmating isolation. The only potential postzygotic isolation factor so far identified is the presence of a migratory divide. If postmating isolation is present in our system, a narrow zone of overlap should be associated with random or near random-mating, and our study provides evidence for this. In order to understand putative reproduction and/or survival consequences in hybrids, we should focus on long-term observations of colour-marked breeding populations in the hybrid zone, allowing for direct assessment of fitness and survival consequences. Experimental approaches that will better allow us to better understand the role and mechanisms of postzygotic factors may include an improved and more robust genotyping protocol containing more subspecies-specific markers that allows the detection and reliable quantification of different genotype classes (e.g. F1, F2, backcrosses). These data could in turn be used to derive more reliable estimates of fitness parameter, survival and reproduction for different genotypes. Further, genomic analyses characterising divergent chromosomal regions and most importantly the identification of candidate genes that are driving the differentiation pattern are likely to be involved in the reproductive isolation and knowledge of their function may help to suggest the mechanism by which reproductive isolation is maintained. In addition to improved genomics resources, future studies will also benefit from more accurate phenotypic measures, e.g. tracking the individuals’ migratory strategy and timing decisions across their annual cycle if technology further advances, especially with relevance for correlating these measures with individual survival and reproduction.

## Materials and methods

### Background

Between 1996 and 2010 over 2000 individuals at 98 sites were sampled across Sweden to investigate trait differences between individuals and populations, and to identify the location and structure of the migratory divide. We focused on three phenotypic traits: plumage colour, morphology (composed of six measurements), and *δ*^15^N (proxy for migratory direction), and one AFLP derived genetic marker whose allele frequency coincides with migratory direction. These data revealed four steep coincident trait clines [[19],[22]], which allowed us to estimate the location of the hybrid zone between 61.4° to 64.1° N (latitude) with the centre of each cline close to 62.7° N [[19]].

### Sampling

Our study was conducted along Flatruet in Sweden (62.7° N, 12.7° E, range 545–846 meters asl, 9 km^2^), within the willow warbler hybrid zone (Additional file 1: Figure S1). In 2011 (10 May to 14 July) and 2012 (2 June to 7 July) we carried out territory mapping and nest searching in order to capture and sample both males and females of all possible breeding pairs in our study population. We captured males with mist-nets by a conspecific song play-back, while the females were captured on or near the nest during the nest-building phase, incubation or while feeding their young. We sampled daily for newly arriving males across the entire study area. This facilitated identification of male arrival dates using capture data as a proxy for the arrival. For conservative estimates of arrival time we only included males caught in 2011, as late winter storms delayed breeding behaviour of arriving males by at least two weeks in 2012.

Each individual was marked with a unique combination of metal and colour rings to facilitate re-sighting and behavioural observation throughout the breeding season. Females, social mates, were all identified by the presence of a brood patch or observation during nest building (carried out solely by females), during incubation or brooding nestlings at the nest attended by their respective male partners. We confirmed both members of all pairs by colour-ring identification or direct (re)capture at the nest.

We followed previous protocols [[1],[19],[22]] to collect four morphometrics (i.e. wing chord, whole tail, tarsus, bill-head), assess plumage colouration, sample blood for subsequent genetic analyses, and collect the first primary in both wings to assess wintering ground *δ*^15^N for all individuals captured. Methods of analyzing *δ*^15^N from feathers have been fully described elsewhere [[1],[19],[35]]. Plumage colour scores were assessed on a scale of 1 to 9 using three reference specimens representing the typical *acredula* (dull grayish-green plumage; score 2), intermediate (score 5) and the typical *trochilus* (yellow plumage; score 8) [[19]]. In an earlier study we analysed sonograms from individuals with the most extreme migratory phenotypes across the Scandinavian distribution range in order to assess if sub-specific song differences (e.g. pattern, repertoire size) could be a factor supporting assortative mating (unpublished results). Because we could not detect any obvious song differences between northern and southern individuals we did not examine this variable as putative factor in assortative mating. Further all captures of males across the Scandinavian distribution range occurred using the same song recording for play-back.

### Genotyping

We developed a genotyping scheme to allow for an assessment of each individual’s genetic ancestry to subspecies, and to identify potential hybrids and backcrosses within the population. Individuals were genotyped for variation in the *NBEA* and *C11orf41* gene, which are located in regions on chromosome 1 (*NBEA*) and chromosome 5 (*C11orf41*); previously identified to be highly differentiated between the subspecies [[24]]. For each gene we manually designed forward and reverse primers in separate exons to amplify polymorphic introns. Primers for NBEA (F: 5’-CCCTCCCAGAAAGAAATCATATCA-3’, R: 5’-TAGCAGCTGCAGCACATCATGAA-3’) were designed from willow warbler 454 Expressed Sequence Tags (ESTs) aligned to zebra finch genome [[24]] and amplified 990 bp of the gene. C11orf41 primers (F: 5’-TCCTGTGGCTGTTGCCTCTC-3’, R: 5’-CCAATATCTGATGTAGACCTGTGC-3’) were designed from an alignment between chicken and zebra finch sequences and amplified 830 bp of the gene.

Amplification was performed using a touchdown PCR starting with an annealing temperature of 63°C, which decreased with 0.5°C per cycle for 12 cycles, followed by 28 cycles with an annealing temperature of 57°C. Each cycle contained an elongation step of 1:15 minutes. PCR reactions were performed in a 25 μl volume consisting of 0.125 mM of each dNTP, 1.5 mM MgCl2, 0.4 μM of primer, 0.5 units of Taq Polymerase (AmpliTaq®, Applied Biosystems, Branchburg, NJ, USA) and 10–50 ng of DNA template. PCR products were quality checked on 2% Agarose Gels and precipitated with 11 μl Ammonium Acetate and 37.5 μl 95% Ethanol. Sequencing was performed using BigDye® Terminator v.1.1 cycle sequencing kit (Applied Biosystems, Austin, TX, USA) on an ABI 3130 prism robot (Applied Biosystems, Foster City, CA, USA).

Sequencing the full length of the PCR products was complicated by the occurrence of multiple length polymorphisms in many individuals. Sequencing efforts were therefore focused on the initial few hundred base pairs of each gene. An internal set of primers (Additional file 1: Table S1) was used for individuals that were heterozygous for multiple length repeats within this interval. Amplification and sequencing using additional primer sets followed the same protocol described above, with the exception of a PCR elongation step of 30 seconds.

Sequences were visualized and trimmed in Geneious Pro 5.6 [[36]]. Alignments of sequences were generated with the Clustal W algorithm [[37]] implemented in Geneious, with manual adjustments when necessary. We manually scored heterozygous positions, and deposited sequence data (of at least 200 bp) in GenBank (Accession numbers: KJ886575-KJ886931, Additional file 2: Table S4). Sequence information for reads <200 bp is provided in Additional file 2: Table S5.

### Population assignment

We used STRUCTURE version 2.3.4 [[30]] to calculate assignment probabilities to subspecies-specific population clusters based on the genotype data for all individuals. Sequences from 15 individuals from northern Sweden and 18 individuals from southern Sweden (Additional file 1: Table S2) were included as reference samples of each subspecies. The input file consisted of single nucleotide polymorphisms (SNPs) with a minor allele frequency of at least 0.10 among the reference samples, which could then be successfully genotyped in the vast majority of the individuals in the full data set. This resulted in five SNPs for *NBEA* and 16 SNPs for *c11orf41*, spanning 181 bp and 341 bp of each gene, respectively.

We ran the software using the admixture model and assuming the existence of two population clusters (K = 2) corresponding to both subspecies. We justify this choice by the existence of two subspecies of the willow warbler in Scandinavia and a hybrid zone between these populations, which has previously been characterised by cline analyses of genetic and phenotypic data in Central Sweden [[19]]. The optimal value of K was also determined from STRUCTURE simulations by running the same analysis for K = 1 to K = 10 with 15 iterations for each K. The results from these runs were analysed using the online tool STRUCTUREharvester [[38]], which implements the K selection method proposed by Evanno et al. [[39]]. In this case, K = 2 also had the highest support (∆K = 1859.83 (K = 2); Second highest K = 3: ∆K = 131.25).

To obtain ancestry estimates for each individual with two population clusters, the admixture model was first ran without explicitly using the samples from the two reference populations to assist clustering of samples at the study site. As expected, the samples from each of the reference populations showed a high assignment probability to each of the two population clusters, respectively. In the final run, we specifically used the reference samples to aid clustering of samples from our study site and used them to update allele frequencies in each of the population clusters throughout the simulation. The model was run with 500 000 burn-ins followed by 500 000 steps of Markov Chain Monte Carlo (MCMC) iterations. We otherwise used default parameters, including the assumption of correlated allele frequencies between the population clusters.

The admixture model assumes that the genetic markers within populations are in linkage equilibrium within each population cluster. This could be violated if the markers are tightly linked to each other, such as polymorphisms within the same gene used in this study. Although STRUCTURE does not specifically model tightly linked genetic markers [[40]], we checked the consistency of the clustering by also analysing the same data set using the linkage model, which is a generalisation of the normal admixture model that takes into account differences in linkage between loci [[41]]. In the model, SNPs located in the different genes were specified as unlinked, and for SNPs within the same gene, nucleotide distances were used as a proxy for genetic distances with an assumed a linear relationship between physical and genetic distance with 1 centimorgan/Mb and used default settings with the exception of a minimum recombination rate prior of 10^−6^. The clustering performed by the linkage model was generally accompanied by slightly more uncertainty, but the individual genetic ancestry estimates were very strongly correlated with those calculated from the admixture model (R = 0.94, p = 2.2 × 10^−16^).

### Statistical analyses

To determine if there were any correlations between male *δ*^15^N values or the probability of northern ancestry to arrival (capture) date, we calculated Pearson’s correlation coefficients (r). We limited our sample of males to those captured in 2011 as the 2012 season started two weeks late due to extended winter conditions. Next, we summarised morphometric data using a principal components analysis where PC1 explained the largest percentage of the variation in our data and was used for subsequent analyses (Additional file 1: Table S3). To determine if there was a relationship between the arrival (capture) dates of males on the breeding grounds we used a Pearson’s correlation analysis. Specifically, we tested capture date against genetic ancestry and difference in migratory strategy for both subspecies. We restricted the analysis with respect to arrival time to males caught in 2011 during the period of 10 May to 10 June, as this represented the period during which we were confident that only newly arrived birds were captured. We further tested for correlations between phenotypic (i.e. plumage colour, PC1 for morphological measurements, *δ*^15^N values) and genotypic (genetic ancestry) traits between social mates using Pearson’s correlation. All statistical analyses were carried out using R [[42]].

## Competing interests

The authors declare that they have no competing interests.

## Authors’ contributions

SB, SÅ, KWL and MLu participated in the study design. KWL, MLu, MLi and AG conducted the field work. MLu and MLi carried out the genetics analyses. KH and LIW carried out the stable isotope analyses. KWL statistically analysed the data. MLi wrote the manuscript. All authors read and approved the final manuscript.

## Additional files

## Supplementary Material

Additional file 1: Figure S1. Study site: Flatruet. **Table S1.** Internal primers used for amplification and sequencing of samples whose genotypes could not be determined by using the standard primers for each locus. **Table S2.** Samples used for STRUCTURE analysis and the resulting assignment probability (genetic ancestry) for each individual to a northern (*acredula*) subspecies population cluster. “Population” indicates individuals caught in the distribution range of the southern (*Phylloscopus trochilus* ***trochilus***) or northern (*Phylloscopus trochilus* ***acredula***) subspecies, or within the hybrid zone. **Table S3.** Eigenvalues and proportion of variance explained by each principal component using four morphological measurements, i.e. wing chord, tail, tarsus, and bill-head length, separately for males and females. Click here for file

Additional file 2: Table S4. Deposited sequence data (of at least 200 bp) in GenBank for polymorphic regions on chromosome 1 (Chr1; NBEA) and chromosome 5 (Chr5; C11orf41). **Table S5.** Sequence data for short reads (< 200 bp) for polymorphic regions on chromosome 1 (Chr1; NBEA). Click here for file
