# Supplementary material for: No evidence for assortative mating within a willow warbler migratory divide
Source: Front Zool. 2014 Jul 12;11:52. doi: 10.1186/s12983-014-0052-2 (PMC4105873; doi:10.1186/s12983-014-0052-2)
Supplement: Additional file 1: Figure S1. — Study site: Flatruet. Table S1. Internal primers used for amplification and sequencing of samples whose genotypes could not be determined by using the standard primers for each locus. Table S2. Samples used for STRUCTURE analysis and the resulting assignment probability (genetic ancestry) for each individual to a northern (acredula) subspecies population cluster. “Population” indicates individuals caught in the distribution range of the southern (Phylloscopus trochilus trochilus ) or northern (Phylloscopus trochilus acredula ) subspecies, or within the hybrid zone. Table S3. Eigenvalues and proportion of variance explained by each principal component using four morphological measurements, i.e. wing chord, tail, tarsus, and bill-head length, separately for males and females. [file s12983-014-0052-2-S1.docx]

**
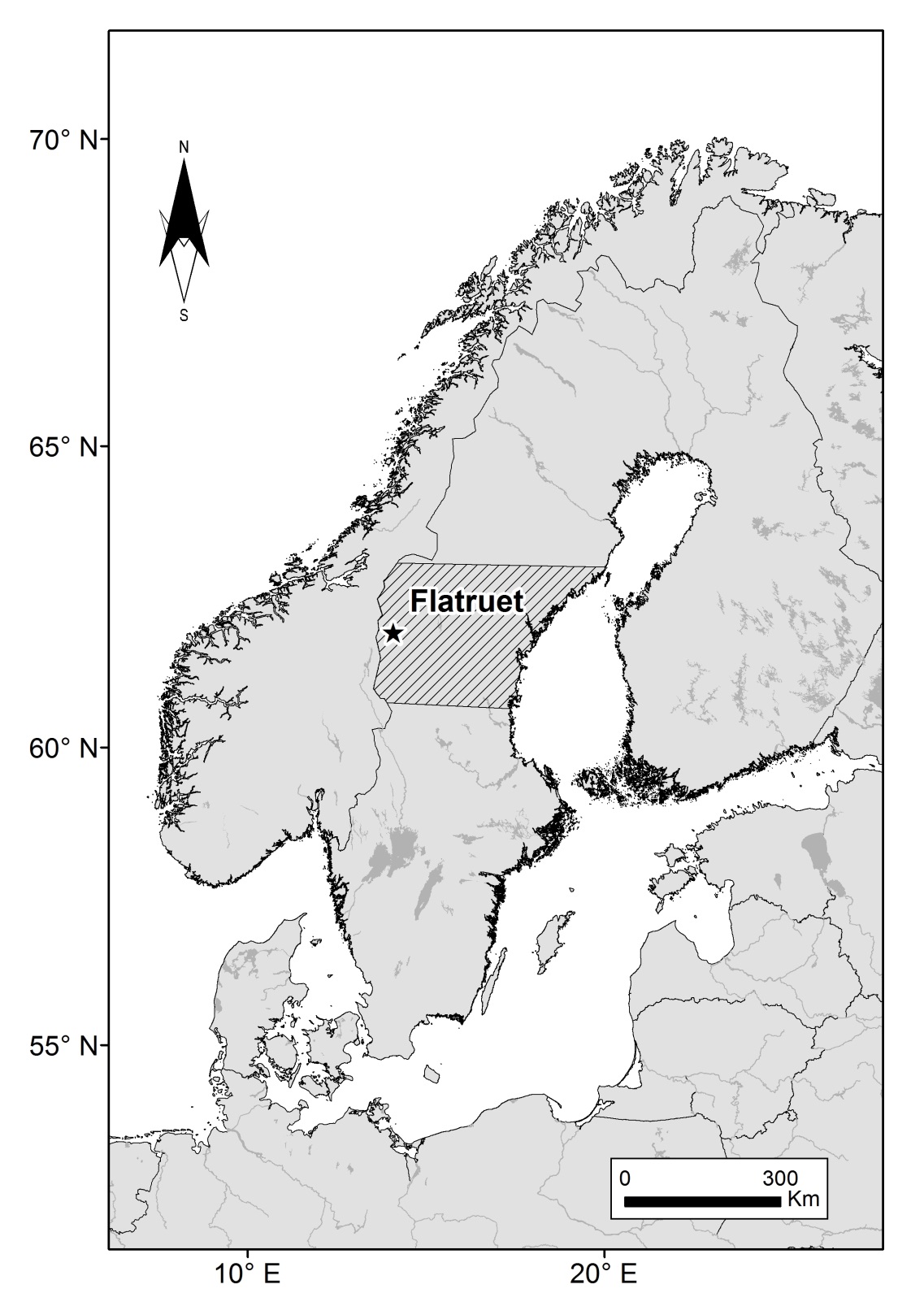
**

Figure S1. **Study site: Flatruet** (62.7° N, 12.7° E, 545-846 meters asl). The cross-hatched area represents the willow warbler hybrid zone across their central Scandinavian migratory divide as described in [19] between 61.4˚ to 64.1˚ N (latitude), where both willow warbler subspecies meet in parapatry.

**Table S1.** Internal primers used for amplification and sequencing of samples whose genotypes could not be determined by using the standard primers for each locus.

| **Primer name** | **Gene** | **Sequence (5'-3')** |
| --- | --- | --- |
| Chr1_52.0MB_4R | *NBEA* | AAATRTAGAYGTCAGAGTTTGGTG |
| Chr5_3.8MB_2F | *C11orf41* | CCTGCTTSCCTGCCTTGTC |
| Chr5_3.8MB_3R | *C11orf41* | CACCASAGCTTCACCACAAYTC |

**Table S2.** Samples used for STRUCTURE analysis and the resulting assignment probability (genetic ancestry) for each individual to a northern (*acredula*) subspecies population cluster. “Population” indicates individuals caught in the distribution range of the southern (*Phylloscopus trochilus* ***trochilus***) or northern (*Phylloscopus trochilus* ***acredula***) subspecies, or within the hybrid zone.

| ID | Sex | Nest Mate | Population | Site | Lat. | Long. | Date | Prob. N |  |
| --- | --- | --- | --- | --- | --- | --- | --- | --- | --- |
| 11D/01 | M |  | *trochilus* | Stensoffa | 55.70 | 13.46 | 29 May 2011 | 0 |  |
| 11D/02 | M |  | *trochilus* | Stensoffa | 55.70 | 13.46 | 02 June 2011 | 0 |  |
| 96A/05 | M |  | *trochilus* | Stensoffa | 55.70 | 13.46 | 07 May 1996 | 0,0066 |  |
| 96A/13 | M |  | *trochilus* | Stensoffa | 55.70 | 13.46 | 10 May 1996 | 0,001 |  |
| 97A/03 | M |  | *trochilus* | Haganäs | 56.25 | 14.67 | 15 May 1997 | 0,0003 |  |
| 97A/05 | M |  | *trochilus* | Haganäs | 56.25 | 14.67 | 15 May 1997 | 0,0067 |  |
| 97B/01 | M |  | *trochilus* | Växjö | 56.83 | 14.73 | 22 May 1997 | 0,0002 |  |
| 97B/10 | M |  | *trochilus* | Växjö | 56.83 | 14.73 | 23 May 1997 | 0,0005 |  |
| 01Q/01 | M |  | *trochilus* | Gräsmarö | 58.28 | 16.98 | 22 June 2001 | 0,0706 |  |
| 01Q/02 | F |  | *trochilus* | Gräsmarö | 58.28 | 16.98 | 23 June 2001 | 0,0197 |  |
| 97E/05 | M |  | *trochilus* | Tåkern | 58.32 | 14.82 | 28 May 1997 | 0 |  |
| 97E/06 | M |  | *trochilus* | Tåkern | 58.32 | 14.82 | 28 May 1997 | 0,0001 |  |
| 00J/01 | M |  | *trochilus* | Smedstorp | 59.58 | 14.98 | 26 June 2000 | 0,002 |  |
| 00J/02 | M |  | *trochilus* | Smedstorp | 59.58 | 14.98 | 26 June 2000 | 0,0009 |  |
| 00H/01 | M |  | *trochilus* | Barkö | 60.27 | 18.27 | 11 June 2000 | 0,2005 |  |
| 00H/02 | M |  | *trochilus* | Barkö | 60.27 | 18.27 | 11 June 2000 | 0 |  |
| 01P/01 | M |  | *trochilus* | Barkö | 60.27 | 18.27 | 14 June 2001 | 0,0001 |  |
| 01P/02 | M |  | *trochilus* | Barkö | 60.27 | 18.27 | 14 June 2001 | 0,0003 |  |
| 11B/01 | M | 11B/35 | hybrid zone | Flatruet | 62.71 | 12.70 | 13 May 2011 | 0,8724 |  |
| 11B/02 | M |  | hybrid zone | Flatruet | 62.71 | 12.70 | 17 May 2011 | 0,1477 |  |
| 11B/03 | M |  | hybrid zone | Flatruet | 62.71 | 12.70 | 20 May 2011 | 0,1798 |  |
| 11B/04 | M | 11B/49 | hybrid zone | Flatruet | 62.71 | 12.70 | 20 May 2011 | 0,0494 |  |
| 11B/05 | M |  | hybrid zone | Flatruet | 62.71 | 12.70 | 20 May 2011 | 0,2851 |  |
| 11B/06 | M |  | hybrid zone | Flatruet | 62.71 | 12.70 | 20 May 2011 | 0,0889 |  |
| 11B/07 | M | 11B/30 | hybrid zone | Flatruet | 62.71 | 12.70 | 20 May 2011 | 0,1518 |  |
| 11B/08 | M | 11B/36 | hybrid zone | Flatruet | 62.71 | 12.70 | 22 May 2011 | 0,0728 |  |
| 11B/09 | M |  | hybrid zone | Flatruet | 62.71 | 12.70 | 23 May 2011 | 0,2087 |  |
| 11B/10 | M |  | hybrid zone | Flatruet | 62.71 | 12.70 | 26 May 2011 | 0,5303 |  |
| 11B/11 | M |  | hybrid zone | Flatruet | 62.71 | 12.70 | 26 May 2011 | 0,2252 |  |
| 11B/12 | M | 11B/47 | hybrid zone | Flatruet | 62.71 | 12.70 | 26 May 2011 | 0,4483 |  |
| 11B/13 | F |  | hybrid zone | Flatruet | 62.71 | 12.70 | 26 May 2011 | 0,4614 |  |
| 11B/14 | M | 11B/50 | hybrid zone | Flatruet | 62.71 | 12.70 | 26 May 2011 | 0,3904 |  |
| 11B/15 | M | 11B/48 | hybrid zone | Flatruet | 62.71 | 12.70 | 30 May 2011 | 0,4362 |  |
| 11B/16 | M | 11B/44 | hybrid zone | Flatruet | 62.71 | 12.70 | 30 May 2011 | 0,5308 |  |
| 11B/17 | M |  | hybrid zone | Flatruet | 62.71 | 12.70 | 30 May 2011 | 0,5243 |  |
| 11B/18 | M |  | hybrid zone | Flatruet | 62.71 | 12.70 | 30 May 2011 | 0,8173 |  |
| 11B/19 | M | 11B/40 | hybrid zone | Flatruet | 62.71 | 12.70 | 30 May 2011 | 0,2842 |  |
| 11B/20 | M |  | hybrid zone | Flatruet | 62.71 | 12.70 | 30 May 2011 | 0,9633 |  |
| 11B/21 | M | 11B/43 | hybrid zone | Flatruet | 62.71 | 12.70 | 30 May 2011 | 0,1887 |  |
| 11B/22 | M | 11B/38 | hybrid zone | Flatruet | 62.71 | 12.70 | 30 May 2011 | 0,4713 |  |
| 11B/24 | M | 11B/52 | hybrid zone | Flatruet | 62.71 | 12.70 | 02 June 2011 | 0,5488 |  |
| 11B/25 | M |  | hybrid zone | Flatruet | 62.71 | 12.70 | 02 June 2011 | 0,1394 |  |
| 11B/26 | M | 11B/53 | hybrid zone | Flatruet | 62.71 | 12.70 | 02 June 2011 | 0,4782 |  |
| 11B/27 | M | 11B/54 | hybrid zone | Flatruet | 62.71 | 12.70 | 02 June 2011 | 0,5722 |  |
| 11B/28 | M |  | hybrid zone | Flatruet | 62.71 | 12.70 | 05 June 2011 | 0,9636 |  |
| 11B/29 | M |  | hybrid zone | Flatruet | 62.71 | 12.70 | 11 June 2011 | 0,7048 |  |
| 11B/30 | F | 11B/07 | hybrid zone | Flatruet | 62.71 | 12.70 | 11 June 2011 | 0,2119 |  |
| 11B/31 | F | 11B/51 | hybrid zone | Flatruet | 62.71 | 12.70 | 14 June 2011 | 0,2392 |  |
| 11B/32 | M | 11B/33 | hybrid zone | Flatruet | 62.71 | 12.70 | 16 June 2011 | 0,0913 |  |
| 11B/33 | F | 11B/32 | hybrid zone | Flatruet | 62.71 | 12.70 | 16 June 2011 | 0,582 |  |
| 11B/34 | F | 11B/23 | hybrid zone | Flatruet | 62.71 | 12.70 | 16 June 2011 | 0,7844 |  |
| 11B/35 | F | 11B/01 | hybrid zone | Flatruet | 62.71 | 12.70 | 02 July 2011 | 0,2692 |  |
| 11B/36 | F | 11B/08 | hybrid zone | Flatruet | 62.71 | 12.70 | 03 July 2011 | 0,4599 |  |
| 11B/37 | F | 11B/45 | hybrid zone | Flatruet | 62.71 | 12.70 | 03 July 2011 | 0,0526 |  |
| 11B/38 | F | 11B/22 | hybrid zone | Flatruet | 62.71 | 12.70 | 03 July 2011 | 0,7214 |  |
| 11B/39 | F | 11B/46 | hybrid zone | Flatruet | 62.71 | 12.70 | 03 July 2011 | 0,111 |  |
| 11B/40 | F | 11B/19 | hybrid zone | Flatruet | 62.71 | 12.70 | 03 July 2011 | 0,5998 |  |
| 11B/41 | F | 11B/42 | hybrid zone | Flatruet | 62.71 | 12.70 | 03 July 2011 | 0,7163 |  |
| 11B/42 | M | 11B/41 | hybrid zone | Flatruet | 62.71 | 12.70 | 03 July 2011 | 0,9241 |  |
| 11B/43 | F | 11B/21 | hybrid zone | Flatruet | 62.71 | 12.70 | 05 July 2011 | 0,2193 |  |
| 11B/44 | F | 11B/16 | hybrid zone | Flatruet | 62.71 | 12.70 | 05 July 2011 | 0,2671 |  |
| 11B/45 | M | 11B/37 | hybrid zone | Flatruet | 62.71 | 12.70 | 06 July 2011 | 0,105 |  |
| 11B/46 | M | 11B/39 | hybrid zone | Flatruet | 62.71 | 12.70 | 06 July 2011 | 0,3149 |  |
| 11B/47 | F | 11B/12 | hybrid zone | Flatruet | 62.71 | 12.70 | 06 July 2011 | 0,0531 |  |
| 11B/48 | F | 11B/15 | hybrid zone | Flatruet | 62.71 | 12.70 | 07 July 2011 | 0,3438 |  |
| 11B/49 | F | 11B/04 | hybrid zone | Flatruet | 62.71 | 12.70 | 07 July 2011 | 0,4114 |  |
| 11B/50 | F | 11B/14 | hybrid zone | Flatruet | 62.71 | 12.70 | 09 July 2011 | 0,0878 |  |
| 11B/51 | M | 11B/31 | hybrid zone | Flatruet | 62.71 | 12.70 | 10 July 2011 | 0,0706 |  |
| 11B/52 | F | 11B/24 | hybrid zone | Flatruet | 62.71 | 12.70 | 12 July 2011 | 0,3142 |  |
| 11B/53 | F | 11B/26 | hybrid zone | Flatruet | 62.71 | 12.70 | 12 July 2011 | 0,3868 |  |
| 11B/54 | F | 11B/27 | hybrid zone | Flatruet | 62.71 | 12.70 | 13 July 2011 | 0,2332 |  |
| 12C/01 | M | 12C/18 | hybrid zone | Flatruet | 62.71 | 12.70 | 05 June 2012 | 0,0809 |  |
| 12C/02 | M |  | hybrid zone | Flatruet | 62.71 | 12.70 | 05 June 2012 | 0,72 |  |
| 12C/03 | M |  | hybrid zone | Flatruet | 62.71 | 12.70 | 05 June 2012 | 0,0449 |  |
| 12C/04 | M | 12C/05 | hybrid zone | Flatruet | 62.71 | 12.70 | 06 June 2012 | 0,2998 |  |
| 12C/05 | F | 12C/04 | hybrid zone | Flatruet | 62.71 | 12.70 | 06 June 2012 | 0,0843 |  |
| 12C/06 | M | 12C/57 | hybrid zone | Flatruet | 62.71 | 12.70 | 06 June 2012 | 0,5293 |  |
| 12C/07 | M | 12C/58 | hybrid zone | Flatruet | 62.71 | 12.70 | 06 June 2012 | 0,2605 |  |
| 12C/08 | F | 12C/09 | hybrid zone | Flatruet | 62.71 | 12.70 | 06 June 2012 | 0,208 |  |
| 12C/09 | M | 12C/08 | hybrid zone | Flatruet | 62.71 | 12.70 | 06 June 2012 | 0,1865 |  |
| 12C/10 | M | 12C/11 | hybrid zone | Flatruet | 62.71 | 12.70 | 06 June 2012 | 0,4896 |  |
| 12C/11 | F | 12C/10 | hybrid zone | Flatruet | 62.71 | 12.70 | 06 June 2012 | 0,3069 |  |
| 12C/12 | M |  | hybrid zone | Flatruet | 62.71 | 12.70 | 06 June 2012 | 0,595 |  |
| 12C/13 | M | 12C/16 | hybrid zone | Flatruet | 62.71 | 12.70 | 07 June 2012 | 0,6395 |  |
| 12C/14 | M |  | hybrid zone | Flatruet | 62.71 | 12.70 | 07 June 2012 | 0,8673 |  |
| 12C/15 | M |  | hybrid zone | Flatruet | 62.71 | 12.70 | 07 June 2012 | 0,4691 |  |
| 12C/16 | F | 12C/13 | hybrid zone | Flatruet | 62.71 | 12.70 | 07 June 2012 | 0,1218 |  |
| 12C/17 | M |  | hybrid zone | Flatruet | 62.71 | 12.70 | 08 June 2012 | 0,2194 |  |
| 12C/18 | F | 12C/01 | hybrid zone | Flatruet | 62.71 | 12.70 | 08 June 2012 | 0,1657 |  |
| 12C/19 | M |  | hybrid zone | Flatruet | 62.71 | 12.70 | 08 June 2012 | 0,0671 |  |
| 12C/20 | M | 12C/50 | hybrid zone | Flatruet | 62.71 | 12.70 | 08 June 2012 | 0,7824 |  |
| 12C/21 | M | 12C/22 | hybrid zone | Flatruet | 62.71 | 12.70 | 08 June 2012 | 0,326 |  |
| 12C/22 | F | 12C/21 | hybrid zone | Flatruet | 62.71 | 12.70 | 08 June 2012 | 0,0861 |  |
| 12C/23 | M | 12C/54 | hybrid zone | Flatruet | 62.71 | 12.70 | 10 June 2012 | 0,0596 |  |
| 12C/24 | M |  | hybrid zone | Flatruet | 62.71 | 12.70 | 10 June 2012 | 0,501 |  |
| 12C/25 | M | 12C/55 | hybrid zone | Flatruet | 62.71 | 12.70 | 10 June 2012 | 0,0935 |  |
| 12C/26 | F | 12C/46 | hybrid zone | Flatruet | 62.71 | 12.70 | 10 June 2012 | 0,0441 |  |
| 12C/27 | M |  | hybrid zone | Flatruet | 62.71 | 12.70 | 10 June 2012 | 0,0679 |  |
| 12C/28 | M |  | hybrid zone | Flatruet | 62.71 | 12.70 | 11 June 2012 | 0,2304 |  |
| 12C/29 | M |  | hybrid zone | Flatruet | 62.71 | 12.70 | 11 June 2012 | 0,2816 |  |
| 12C/30 | F | 12C/31 | hybrid zone | Flatruet | 62.71 | 12.70 | 11 June 2012 | 0,4599 |  |
| 12C/31 | M | 12C/30 | hybrid zone | Flatruet | 62.71 | 12.70 | 11 June 2012 | 0,7585 |  |
| 12C/32 | M |  | hybrid zone | Flatruet | 62.71 | 12.70 | 11 June 2012 | 0,4306 |  |
| 12C/33 | M |  | hybrid zone | Flatruet | 62.71 | 12.70 | 12 June 2012 | 0,467 |  |
| 12C/34 | M |  | hybrid zone | Flatruet | 62.71 | 12.70 | 12 June 2012 | 0,4781 |  |
| 12C/35 | M |  | hybrid zone | Flatruet | 62.71 | 12.70 | 12 June 2012 | 0,4585 |  |
| 12C/36 | F |  | hybrid zone | Flatruet | 62.71 | 12.70 | 13 June 2012 | 0,537 |  |
| 12C/37 | F | 12C/38 | hybrid zone | Flatruet | 62.71 | 12.70 | 13 June 2012 | 0,0529 |  |
| 12C/38 | M | 12C/37 | hybrid zone | Flatruet | 62.71 | 12.70 | 13 June 2012 | 0,0761 |  |
| 12C/39 | M |  | hybrid zone | Flatruet | 62.71 | 12.70 | 14 June 2012 | 0,5295 |  |
| 12C/40 | F |  | hybrid zone | Flatruet | 62.71 | 12.70 | 14 June 2012 | 0,8679 |  |
| 12C/41 | F | 12C/56 | hybrid zone | Flatruet | 62.71 | 12.70 | 15 June 2012 | 0,1665 |  |
| 12C/42 | M |  | hybrid zone | Flatruet | 62.71 | 12.70 | 15 June 2012 | 0,4578 |  |
| 12C/43 | F |  | hybrid zone | Flatruet | 62.71 | 12.70 | 15 June 2012 | 0,0926 |  |
| 12C/44 | M |  | hybrid zone | Flatruet | 62.71 | 12.70 | 15 June 2012 | 0,1814 |  |
| 12C/45 | M |  | hybrid zone | Flatruet | 62.71 | 12.70 | 17 June 2012 | 0,7592 |  |
| 12C/46 | M | 12C/26 | hybrid zone | Flatruet | 62.71 | 12.70 | 17 June 2012 | 0,4315 |  |
| 12C/47 | M |  | hybrid zone | Flatruet | 62.71 | 12.70 | 18 June 2012 | 0,3735 |  |
| 12C/48 | M |  | hybrid zone | Flatruet | 62.71 | 12.70 | 21 June 2012 | 0,3224 |  |
| 12C/49 | M |  | hybrid zone | Flatruet | 62.71 | 12.70 | 21 June 2012 | 0,5074 |  |
| 12C/50 | F | 12C/20 | hybrid zone | Flatruet | 62.71 | 12.70 | 28 June 2012 | 0,5048 |  |
| 12C/54 | F | 12C/23 | hybrid zone | Flatruet | 62.71 | 12.70 | 28 June 2012 | 0,1168 |  |
| 12C/55 | F | 12C/25 | hybrid zone | Flatruet | 62.71 | 12.70 | 28 June 2012 | 0,4932 |  |
| 12C/56 | M | 12C/41 | hybrid zone | Flatruet | 62.71 | 12.70 | 06 July 2012 | 0,5538 |  |
| 12C/57 | F | 12C/06 | hybrid zone | Flatruet | 62.71 | 12.70 | 07 July 2012 | 0,6413 |  |
| 12C/58 | F | 12C/07 | hybrid zone | Flatruet | 62.71 | 12.70 | 07 Aug 2012 | 0,1112 |  |
| 11A/01 | M | 11A/39 | hybrid zone | Flatruet | 62.84 | 12.97 | 11 May 2011 | 0,6942 |  |
| 11A/02 | M |  | hybrid zone | Flatruet | 62.84 | 12.97 | 11 May 2011 | 0,176 |  |
| 11A/03 | M |  | hybrid zone | Flatruet | 62.84 | 12.97 | 12 May 2011 | 0,1535 |  |
| 11A/04 | M |  | hybrid zone | Flatruet | 62.84 | 12.97 | 12 May 2011 | 0,3675 |  |
| 11A/05 | M |  | hybrid zone | Flatruet | 62.84 | 12.97 | 14 May 2011 | 0,1702 |  |
| 11A/06 | M |  | hybrid zone | Flatruet | 62.84 | 12.97 | 14 May 2011 | 0,4092 |  |
| 11A/07 | M |  | hybrid zone | Flatruet | 62.84 | 12.97 | 16 May 2011 | 0,5284 |  |
| 11A/08 | M | 11A/25 | hybrid zone | Flatruet | 62.84 | 12.97 | 16 May 2011 | 0,0508 |  |
| 11A/09 | F | 11A/10 | hybrid zone | Flatruet | 62.84 | 12.97 | 19 May 2011 | 0,7668 |  |
| 11A/10 | M | 11A/09 | hybrid zone | Flatruet | 62.84 | 12.97 | 19 May 2011 | 0,949 |  |
| 11A/11 | M |  | hybrid zone | Flatruet | 62.84 | 12.97 | 19 May 2011 | 0,0642 |  |
| 11A/12 | M |  | hybrid zone | Flatruet | 62.84 | 12.97 | 19 May 2011 | 0,2688 |  |
| 11A/13 | M |  | hybrid zone | Flatruet | 62.84 | 12.97 | 21 May 2011 | 0,4575 |  |
| 11A/14 | M |  | hybrid zone | Flatruet | 62.84 | 12.97 | 21 May 2011 | 0,1948 |  |
| 11A/15 | M |  | hybrid zone | Flatruet | 62.84 | 12.97 | 21 May 2011 | 0,062 |  |
| 11A/16 | M |  | hybrid zone | Flatruet | 62.84 | 12.97 | 21 May 2011 | 0,0534 |  |
| 11A/17 | F |  | hybrid zone | Flatruet | 62.84 | 12.97 | 23 May 2011 | 0,2563 |  |
| 11A/18 | M |  | hybrid zone | Flatruet | 62.84 | 12.97 | 23 May 2011 | 0,2302 |  |
| 11A/19 | M |  | hybrid zone | Flatruet | 62.84 | 12.97 | 26 May 2011 | 0,6475 |  |
| 11A/20 | M |  | hybrid zone | Flatruet | 62.84 | 12.97 | 26 May 2011 | 0,5646 |  |
| 11A/21 | M |  | hybrid zone | Flatruet | 62.84 | 12.97 | 26 May 2011 | 0,2496 |  |
| 11A/22 | M |  | hybrid zone | Flatruet | 62.84 | 12.97 | 28 May 2011 | 0,7306 |  |
| 11A/23 | M |  | hybrid zone | Flatruet | 62.84 | 12.97 | 29 May 2011 | 0,4874 |  |
| 11A/24 | M |  | hybrid zone | Flatruet | 62.84 | 12.97 | 29 May 2011 | 0,9321 |  |
| 11A/25 | F | 11A/08 | hybrid zone | Flatruet | 62.84 | 12.97 | 01 June 2011 | 0,582 |  |
| 11A/26 | M |  | hybrid zone | Flatruet | 62.84 | 12.97 | 01 June 2011 | 0,2028 |  |
| 11A/27 | M | 11A/34 | hybrid zone | Flatruet | 62.84 | 12.97 | 01 June 2011 | 0,6979 |  |
| 11A/28 | M |  | hybrid zone | Flatruet | 62.84 | 12.97 | 02 June 2011 | 0,7011 |  |
| 11A/29 | M |  | hybrid zone | Flatruet | 62.84 | 12.97 | 03 June 2011 | 0,0955 |  |
| 11A/30 | M | 11A/35 | hybrid zone | Flatruet | 62.84 | 12.97 | 03 June 2011 | 0,1476 |  |
| 11A/31 | M |  | hybrid zone | Flatruet | 62.84 | 12.97 | 03 June 2011 | 0,3746 |  |
| 11A/32 | M |  | hybrid zone | Flatruet | 62.84 | 12.97 | 03 June 2011 | 0,1169 |  |
| 11A/33 | F |  | hybrid zone | Flatruet | 62.84 | 12.97 | 06 June 2011 | 0,3845 |  |
| 11A/34 | F | 11A/27 | hybrid zone | Flatruet | 62.84 | 12.97 | 07 June 2011 | 0,4328 |  |
| 11A/35 | F | 11A/30 | hybrid zone | Flatruet | 62.84 | 12.97 | 07 June 2011 | 0,9122 |  |
| 11A/36 | M |  | hybrid zone | Flatruet | 62.84 | 12.97 | 12 June 2011 | 0,4085 |  |
| 11A/37 | M |  | hybrid zone | Flatruet | 62.84 | 12.97 | 12 June 2011 | 0,0657 |  |
| 11A/38 | M |  | hybrid zone | Flatruet | 62.84 | 12.97 | 15 June 2011 | 0,0555 |  |
| 11A/39 | F | 11A/01 | hybrid zone | Flatruet | 62.84 | 12.97 | 06 July 2011 | 0,5063 |  |
| 12B/01 | F |  | hybrid zone | Flatruet | 62.84 | 12.97 | 02 June 2012 | 0,8097 |  |
| 97K/07 | M |  | *acredula* | Klimpfjäll | 65.03 | 14.68 | 15 June 1997 | 0,5754 |  |
| 01O/01 | M |  | *acredula* | Kallax | 65.53 | 22.13 | 12 June 2001 | 0,9976 |  |
| 01O/02 | M |  | *acredula* | Kallax | 65.53 | 22.13 | 12 June 2001 | 0,9833 |  |
| 97M/01 | M |  | *acredula* | Kukkola | 65.96 | 24.03 | 22 June 1997 | 0,9995 |  |
| 97M/02 | M |  | *acredula* | Kukkola | 65.96 | 24.03 | 22 June 1997 | 0,9999 |  |
| 97N/13 | M |  | *acredula* | Gällivare | 67.22 | 20.80 | 27 June 1997 | 0,9999 |  |
| 97N/14 | M |  | *acredula* | Gällivare | 67.22 | 20.80 | 27 June 1997 | 1 |  |
| 01N/01 | M |  | *acredula* | Altajärvi | 67.83 | 20.53 | 12 June 2001 | 0,9985 |  |
| 01N/02 | M |  | *acredula* | Altajärvi | 67.83 | 20.53 | 12 June 2001 | 0,9995 |  |
| 01M/05 | M |  | *acredula* | Kaisepakte | 68.28 | 19.32 | 10 June 2001 | 0,9996 |  |
| 01M/06 | M |  | *acredula* | Kaisepakte | 68.28 | 19.32 | 10 June 2001 | 0,9999 |  |
| 01L/19 | M |  | *acredula* | Stordalen | 68.33 | 19.10 | 11 June 2001 | 1 |  |
| 01L/20 | M |  | *acredula* | Stordalen | 68.33 | 19.10 | 11 June 2001 | 0,9998 |  |
| 01K/01 | M |  | *acredula* | Torneham | 68.43 | 18.58 | 09 June 2001 | 0,7373 |  |
| 01K/02 | M |  | *acredula* | Torneham | 68.43 | 18.58 | 09 June 2001 | 0,9999 |  |

Table S3. Eigenvalues and proportion of variance explained by each principal component using four morphological measurements, i.e. wing chord, tail, tarsus, and bill-head length, separately for males and females.

| ***Males*** | **PC1** | **PC2** | **PC3** | **PC4** |
| --- | --- | --- | --- | --- |
| Wing | -0.61 | 0.21 | 0.29 | 0.71 |
| Tail | -0.61 | 0.09 | 0.36 | -0.70 |
| Tarsus | -0.24 | -0.97 | -0.02 | 0.08 |
| Bill-Head | -0.44 | 0.12 | -0.89 | -0.06 |
| ***Importance*** |  |  |  |  |
| Standard deviation | 1.44 | 0.97 | 0.86 | 0.50 |
| Proportion of Variance | 0.52 | 0.23 | 0.19 | 0.06 |
| Cumulative Proportion | 0.52 | 0.75 | 0.94 | 1.00 |
| ***Females*** | **PC1** | **PC2** | **PC3** | **PC4** |
| Wing | 0.67 | -0.26 | -0.22 | -0.66 |
| Tail | 0.63 | -0.26 | 0.43 | 0.59 |
| Tarsus | 0.36 | 0.59 | -0.64 | 0.34 |
| Bill-Head | 0.18 | 0.72 | 0.60 | -0.30 |
| ***Importance*** |  |  |  |  |
| Standard deviation | 1.22 | 1.07 | 0.93 | 0.71 |
| Proportion of Variance | 0.37 | 0.29 | 0.22 | 0.13 |
| Cumulative Proportion | 0.37 | 0.66 | 0.87 | 1.00 |

Table S4. Willow warbler capture date (day/month/year) trait data, i.e. plumage colour, *δ*^15^N, genetic ancestry to a northern subspecies (*P. t. acredula*) cluster (Prob N; inferred from STRUCTURE analyses), and PC1 for morphological measurements, for all pairs (n=40).

| Male | | | | | | Female | | | | | |
| --- | --- | --- | --- | --- | --- | --- | --- | --- | --- | --- | --- |
| ID | **Captured** | **Colour** | **δ^15^N (‰)** | **Prob N** | **Body Size** (PC1) | **ID** | **Captured** | **Colour** | **δ^15^N (‰)** | **Prob N** | **Body Size** (PC1) |
| 11A/01 | 5/11/2011 | 7 | 6.1 | 0.50 | -3.78 | **11A/39** | 7/6/2011 | 9 | 7.5 | 0.50 | -0.82 |
| 11A/08 | 5/16/2011 | 6 | 6.4 | 0.19 | -2.62 | **11A/25** | 6/1/2011 | 8 | 8.3 | 0.50 | 0.99 |
| 11A/10 | 5/19/2011 | 4 | 7.6 | 0.82 | -1.10 | **11A/09** | 5/19/2011 | 8 | 4.6 | 0.61 | -0.89 |
| 11A/27 | 6/1/2011 | 6 | 7.0 | 0.51 | -0.33 | **11A/34** | 6/7/2011 | 9 | 8.6 | 0.50 | -0.34 |
| 11A/30 | 6/3/2011 | 8 | 8.8 | 0.34 | -0.52 | **11A/35** | 6/7/2011 | 3 | 8.0 | 0.81 | 1.10 |
| 11B/01 | 5/13/2011 | 8 | 10.6 | 0.66 | -0.83 | **11B/35** | 7/2/2011 | 6 | 6.7 | 0.34 | 0.78 |
| 11B/04 | 5/20/2011 | 5 | 7.9 | 0.19 | 0.07 | **11B/49** | 7/7/2011 | 8 | 6.8 | 0.46 | NA |
| 11B/07 | 5/20/2011 | 6 | 7.5 | 0.34 | 0.56 | **11B/30** | 6/11/2011 | 5 | 8.0 | 0.36 | 0.92 |
| 11B/08 | 5/22/2011 | 6 | 6.8 | 0.19 | -0.67 | **11B/36** | 7/3/2011 | 8 | 9.0 | 0.50 | -0.60 |
| 11B/12 | 5/26/2011 | 8 | 7.9 | 0.50 | 0.44 | **11B/47** | 7/6/2011 | 9 | 7.6 | 0.18 | -0.25 |
| 11B/14 | 5/26/2011 | 6 | 8.0 | 0.34 | -0.23 | **11B/50** | 7/9/2011 | 7 | 7.0 | 0.18 | 1.31 |
| 11B/15 | 5/30/2011 | 6 | 7.7 | 0.50 | 0.28 | **11B/48** | 7/7/2011 | 3 | 6.6 | 0.45 | 0.35 |
| 11B/16 | 5/30/2011 | 5 | 10.2 | 0.50 | -0.34 | **11B/44** | 7/5/2011 | 6 | 6.4 | 0.50 | -4.23 |
| 11B/19 | 5/30/2011 | 7 | 7.7 | 0.34 | 0.24 | **11B/40** | 7/3/2011 | 5 | 8.6 | 0.66 | 2.02 |
| 11B/21 | 5/30/2011 | 9 | 7.3 | 0.34 | 0.42 | **11B/43** | 7/5/2011 | 8 | 8.9 | 0.34 | -0.65 |
| 11B/22 | 5/30/2011 | 5 | 7.3 | 0.49 | -0.85 | **11B/38** | 7/3/2011 | 9 | 7.1 | 0.50 | 0.13 |
| 11B/23 | 5/30/2011 | 8 | 6.7 | 0.34 | 1.00 | **11B/34** | 6/16/2011 | 7 | 9.6 | 0.66 | 1.50 |
| 11B/24 | 6/2/2011 | 7 | 8.0 | 0.51 | -1.51 | **11B/52** | 7/12/2011 | 3 | 6.1 | 0.45 | 1.95 |
| 11B/26 | 6/2/2011 | 5 | 7.5 | 0.50 | 2.73 | **11B/53** | 7/12/2011 | 6 | 6.7 | 0.34 | -0.33 |
| 11B/27 | 6/2/2011 | 5 | 12.1 | 0.66 | -3.47 | **11B/54** | 7/13/2011 | 4 | 8.1 | 0.34 | -0.78 |
| 11B/32 | 6/16/2011 | 4 | 7.1 | 0.20 | 3.45 | **11B/33** | 6/16/2011 | 4 | 6.5 | 0.66 | -0.96 |
| 11B/42 | 7/3/2011 | 6 | 8.9 | 0.79 | 0.61 | **11B/41** | 7/3/2011 | 4 | 9.0 | 0.50 | -1.32 |
| 11B/45 | 7/6/2011 | 7 | 9.6 | 0.29 | 0.24 | **11B/37** | 7/3/2011 | 8 | 7.0 | 0.18 | -1.12 |
| 11B/46 | 7/6/2011 | 7 | 5.6 | 0.34 | 0.68 | **11B/39** | 7/3/2011 | 8 | 5.1 | 0.20 | -0.05 |
| 11B/51 | 7/10/2011 | 3 | 7.2 | 0.18 | 0.65 | **11B/31** | 6/14/2011 | 6 | 12.8 | 0.32 | 0.08 |
| 12C/01 | 6/5/2012 | 6 | 7.9 | 0.19 | 0.24 | **12C/18** | 6/8/2012 | 7 | 8.5 | 0.34 | -0.37 |
| 12C/04 | 6/6/2012 | 5 | 9.1 | 0.34 | 1.65 | **12C/05** | 6/6/2012 | 4 | 11.3 | 0.18 | 0.72 |
| 12C/06 | 6/6/2012 | 4 | 7.9 | 0.34 | -0.92 | **12C/57** | 7/7/2012 | 2 | 8.2 | 0.50 | -2.45 |
| 12C/07 | 6/6/2012 | 7 | 11.8 | 0.34 | -0.24 | **12C/58** | 7/7/2012 | 8 | 5.9 | 0.20 | -0.47 |
| 12C/09 | 6/6/2012 | 4 | 10.5 | 0.34 | 0.11 | **12C/08** | 6/6/2012 | 9 | 8.4 | 0.34 | 0.96 |
| 12C/10 | 6/6/2012 | 3 | 7.9 | 0.50 | 0.38 | **12C/11** | 6/6/2012 | 5 | 6.1 | 0.34 | -0.31 |
| 12C/13 | 6/7/2012 | 9 | 12.2 | 0.66 | 1.49 | **12C/16** | 6/7/2012 | 6 | 9.1 | 0.19 | -1.31 |
| 12C/20 | 6/8/2012 | 4 | 5.2 | 0.66 | -0.51 | **12C/50** | 6/28/2012 | 8 | 7.6 | 0.35 | 0.12 |
| 12C/21 | 6/8/2012 | 4 | 7.8 | 0.34 | -1.14 | **12C/22** | 6/8/2012 | 8 | 7.7 | 0.19 | 1.34 |
| 12C/23 | 6/10/2012 | 4 | 7.5 | 0.19 | -0.07 | **12C/54** | 6/28/2012 | 4 | 6.6 | 0.19 | 0.42 |
| 12C/25 | 6/10/2012 | 4 | 10.6 | 0.19 | -0.98 | **12C/55** | 6/28/2012 | 4 | 7.1 | 0.65 | -0.70 |
| 12C/31 | 6/11/2012 | 9 | 6.8 | 0.50 | 2.67 | **12C/30** | 6/11/2012 | 5 | 9.9 | 0.50 | 0.07 |
| 12C/38 | 6/13/2012 | 6 | 7.2 | 0.19 | 1.75 | **12C/37** | 6/13/2012 | 9 | 7.1 | 0.19 | 0.89 |
| 12C/46 | 6/17/2012 | 6 | 7.4 | 0.50 | 0.39 | **12C/26** | 6/10/2012 | 7 | 9.8 | 0.18 | 0.81 |
| 12C/56 | 7/6/2012 | 6 | 7.0 | 0.50 | 0.08 | **12C/41** | 6/15/2012 | 8 | 6.9 | 0.34 | 1.46 |
